# GnRH agonists to sustain the luteal phase in antagonist IVF cycles: a randomized prospective trial

**DOI:** 10.1186/s12958-019-0543-2

**Published:** 2019-11-29

**Authors:** Francesco M. Fusi, Claudio M. Brigante, Laura Zanga, Mario Mignini Renzini, Chiara Bosisio, Rubens Fadini

**Affiliations:** 1 ASST Papa Giovanni XXIII, Piazza OMS 1, 24127 Bergamo, Italy; 2 Università degli Studi Bicocca, Milan, Italy; 3 Biogenesi Reproductive Medicine Center , Istituti Clinici Zucchi, Monza, Italy

**Keywords:** GnRH antagonist, Luteal phase, Triptorelin, IVF, GnRH agonist

## Abstract

**Background:**

The addition of a GnRH analogue to the luteal phase in in vitro fertilization programs has been seldom proposed due to the presence of GnRH receptors in the endometrium. The aim of the study was to evaluate the effect of triptorelin addition in short antagonist cycles, compared to cycles where the only supplementation was progesterone.

**Methods:**

The primary objective of this study was the study of the effect of Triptorelin addiction during the luteal phase on the live birth rate. Secondary objectives of efficacy were pregnancy rates and implantation rates, as well as safety in terms of OHSS risks. The study was a prospective, randomized, open study, performed in two independent Centers from July 2013 to October 2015. Patients were divided into three groups: a) Regular antagonist protocol, with only luteal progesterone; b) Antagonist protocol with luteal triptorelin as multiple injections, c) Antagonist protocol with luteal triptorelin as single bolus. Descriptive statistics were obtained for all the parameters. Mean and standard deviation were used for all quantitative parameters. Differences between percentages were studied using Chi-square test generalized to the comparison of several proportions.

**Results:**

A total number of 1344 patients completed the study, 786 under the age of 35 years, and 558 over 35 years. It was observed an increase of positive HCG results, Clinical pregnancy rates and Delivery rates when triptorelin was added in the luteal phase, irrespective whether as a single bolus or five injections. This increase was statistically significant both for pregnancy rates and delivery rates. The statistic difference between pregnancies and deliveries obtained with or without luteal triptorelin reached *p* < 0,01. No increase of OHSS risk was observed.

**Conclusions:**

From this large study it appears that the concept of luteal phase supplementation should be revisited. From our study it appears that triptorelin addition to the luteal phase of antagonist cycles, either as a single bolus or using multiple injections, is a good tool to optimize ART results.

**Trial registration:**

The study was approved by the Ethics Committee of Provincia di Bergamo (n 1203/2013).

## Background

The luteal phase supplementation was reported to be necessary in controlled hyperstimulation cycles for IVF or ICSI, independently when GnRH agonists or antagonists were used as pituitary desensitization. Progesterone is commonly used as main luteal phase support in all cycles, and for many years no different approaches have been attempted. The introduction of GnRH antagonists in the common clinical practice of in vitro fertilization (IVF) cycles has raised several new concepts, both for the trigger to be used, and for the attention to the luteal phase [1, 2].

Tesarik et al. have shown for the first time that the luteal phase GnRH agonist administration enhances clinical outcomes after GnRH agonist and GnRH antagonist-treated ovarian stimulation cycles [3, 4]. It has been suggested that GnRH may act both through an indirect stimulus to corpora lutea by gonadotropin discharge from pituitary gland, leading to a stimulus to corpora lutea, and via a direct effect on endometrium and embryo [5]. The data on donor cycles, obtained when triptorelin was added in the luteal phase in the absence of corpora lutea, suggested that the effect might be due to a direct effect, on the endometrium or the embryo [3, 6].

Starting from these data it has been postulated that the luteal phase support exclusively with progesterone might not always be sufficient to promote implantation, and other approaches can be attempted [3, 4, 7].

Several ways to use a GnRH analogue have been proposed: triptorelin can be administered as a single bolus 1 week after the oocyte retrieval [6, 7], or 0,1 mg triptorelin can be given every other day from the day of embryo transfer for a total of five injections [8]. In alternative, a low dose of buserelin spray can be given daily for 2 weeks during the luteal phase [9–11]. At any rate, the statistic power of these results was limited by the small number and different characteristics of the enrolled patients, and by the actual design of the studies, most of them being observational.

The study we are presenting was a prospective, randomized study, performed in two independent Centers. The aim of the study was to evaluate the effect of triptorelin addition, as a single bolus or five injections, in short antagonist cycles with HCG trigger.

## Materials and methods

### Objectives

The primary objective of this study was the study of the effect of Triptorelin addiction during the luteal phase on the live birth rate. Secondary objectives of efficacy were pregnancy rates and implantation rates, as well as safety in terms of OHSS risks.

### Patient selection

Patients were recruited in two independent Centers for Reproductive Medicine, according to the following criteria:

#### Inclusion criteria

- Age < 40 years
- AFC (Antral Follicular Count) > 5 < 15
- AMH (Anti-Mullerian Hormone) > 1,5
- FSH (Follicle Stimulating Hormone) < 10
- Regular menses

#### Exclusion criteria

- Endometriosis III o IV stage
- Severe male factor (cryptozoospermia or azoospermia)
- PCOs (Polycystic ovary syndrome)
- BMI (Body mass Index) < 18 or > 30
- Non-balanced Thyroid disfunction

Informed consent was obtained from all individual participants included in the study.

### Study design

The study was a prospective, randomized, open study, performed in two independent Centers from July 2013 to October 2015. For randomization, the criterion of allocation to each arm of the treatment was a computer-generated randomization sheet of the patients fulfilling the inclusion criteria. Patients were recruited in a ratio 1: 1 respectively for group A (controls), B (luteal triptorelin in five doses) and C (luteal administration of a single bolus of triptorelin). The study was approved by the Local Ethics Committee (n 1203/2013).

### Treatment

Patients were divided into the following groups:

A. Antagonist protocol (AH). r-FSH 150–225 UI/day was given from day 3 of the cycle. GnRH antagonist was added when leading follicle was 13 mm. Final trigger was performed using r-HCG 6000 UI or HCG 10000 UI. Luteal phase was supported using vaginal progesterone, 600 mg/day.
B. Antagonist protocol with luteal multiple administrations of triptorelin (AHT1). R-FSH 150–225 UI/day was given from day 3 of the cycle. GnRH antagonist was added when leading follicle was 13 mm. Final trigger was performed using r-HCG 6000 UI or HCG 10000 UI. Luteal phase was supported using vaginal progesterone, 600 mg/day. In addition Triptorelin 0,1 mg was given starting from the day of embryo transfer every other day to a total of five injections.
C. Antagonist protocol with luteal Triptorelin a a single bolus (AHT2). In this group of patients, the treatment was the same as group b, except that Triptorelin was given as a single 0,2 mg injection in day 6 after oocyte collection.

In all the groups Embryo transfer was performed on day three. Beta HCG was determined at 12 days from embryo transfer.

In the case of OHSS risk at the time of final trigger, patients were excluded from the study. The safety of triptorelin addition in this study was evaluated on the late OHSS onset.

### Statistical analysis

Descriptive statistics were obtained for all the parameters. Mean and standard deviation were used for all quantitative parameters. Differences between percentages were studied using Chi-square test generalized to the comparison of several proportions. The minimal number of cases to establish statistical significance was calculated to be 800 in total.

## Results

The patients who were randomized were in total 1367. A total number of 1344 patients completed the study, 786 under the age of 35 years, and 558 over 35 years.

No differences were observed between treatment groups in the charactesistics of the patients enrolled (Table 1), number of retrieved oocytes, inseminated oocytes, embryos obtained and embryos transferred (Table 2).

**Table 1:** Comparison of patient’s characteristics between groups of treatment

	AH	AHT1	AHT2	
Mean age group < 35	33,4 ± 1,7	33,7 ± 1,3	32,8 ± 1,7	NS
Mean age group ≥35	38,6 ± 2,3	38,1 ± 2,4	38,9 ± 2,1	NS
% nulliparous	73,4%	71,8%	74,1%	NS
% male factor	24,9%	27,1%	22,6%	NS
% tubal factor	12,3%	12,7%	14,0%	NS
% mixed factor or no factor	62,8%	60,2%	63,4%	NS
Mean BMI group < 35	22,3 ± 2,8	23,1 ± 2,6	22,1 ± 3,3	NS
Mean BMI group > 35	24,3 ± 3,7	24,2 ± 3,4	24,5 ± 3,5	NS

*AH* Antagonist cycles with only Progesterone in luteal phase

*AHT1* Antagonist cycles with addition of luteal triptorelin as multiple injections

*AHT2* Antagonist cycles with addition of luteal triptorelin as single bolus

**TABLE 2:** Laboratory data from different groups

		AH	AHT1	AHT2	
Retrieved oocytes (Mean ± SD)	**< 35 anni**	**8,7** ± **4,5**	**9,2** ± **5,3**	**9,3** ± **4,2**	**NS**
	**≥35 anni**	**7,8** ± **4,1**	**7,6** ± **4,3**	**7,3** ± **2,9**	**NS**
Inseminated oocytes (Mean ± SD)	**< 35 anni**	**5,2** ± **2,1**	**5,3** ± **2,4**	**4,2** ± **1,4**	**NS**
	**≥35 anni**	**5,7** ± **2,9**	**5,9** ± **2,6**	**6,1** ± **2,4**	**NS**
Embryos obtained (Mean ± SD)	**< 35 anni**	**3,9** ± **1,8**	**3,9** ± **1,6**	**3,1** ± **0,9**	**NS**
	**≥35 anni**	**4,1** ± **2,2**	**4,0** ± **2,0**	**4,2** ± **2,1**	**NS**
Transferred embryos (Mean ± SD)	**< 35 anni**	**1,7** ± **0,6**	**1,7** ± **0,6**	**1,5** ± **0,7**	**NS**
	**≥35 anni**	**2,2** ± **0,6**	**2,2** ± **0,7**	**2,3** ± **0,7**	**NS**

*AH* Antagonist cycles with only Progesterone in luteal phase

*AHT1* Antagonist cycles with addition of luteal triptorelin as multiple injections

*AHT2* Antagonist cycles with addition of luteal triptorelin as single bolus

**Fig. 1:**
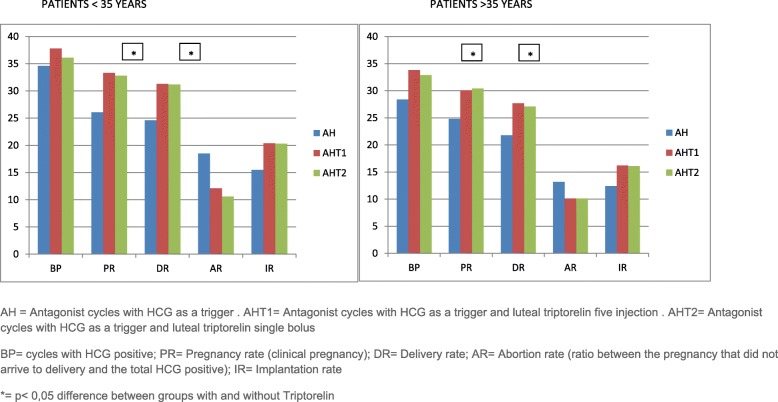
Comparison of results when no luteal triptorelin or different regimens of luteal triptorelin were given. AH = Antagonist cycles with HCG as a trigger . AHT1 = Antagonist cycles with HCG as a trigger and luteal triptorelin five injection . AHT2 = Antagonist cycles with HCG as a trigger and luteal triptorelin single bolus. BP = cycles with HCG positive; PR = Pregnancy rate (clinical pregnancy); DR = Delivery rate; AR = Abortion rate (ratio between the pregnancy that did not arrive to delivery and the total HCG positive); IR = Implantation rate. * = *p* < 0,05 difference between groups with and without Triptorelin.

As shown in Fig. 1 it was observed an increase of positive HCG results, clinical pregnancy rates and Delivery rates when triptorelin was added in the luteal phase, either when triptorelin was added as a single bolus or as repeated injections. In particular, cycles with positive beta HCG were respectively 37,85 and 36,1% using Triptorelin five injections or single bolus in patients < 35 years of age, 33,8% and 32,9% in patients over 35. The positive beta were 34,6% and 28,4% in < 35 and > 35 in the control group (group A, no luteal triptorelin). For this parameter there was an increase, but it did not reach a statistic significance. Conversely, ongoing pregnancy rates and delivery rates reached a significance of *p* < 0,05 for both subgroups with luteal triptorelin compared to the control, either in patients under 35 (33,3% and 32,8% versus 26,1% for Pregnancy rate; 31,3% and 31,2% a versus 24,6% for Delivery rate) or in patients over 35 (30,1% and 30,4% versus 24,8% for Pregnancy rate; 27,7% and 27,1% versus 21,8% for Delivery rate).

No differences were observed when Triptorelin was added as a single bolus or multiple injections. For this reason, we decided to cumulate the data from the two groups that received luteal triptorelin, maintaining the two categories of patients, < 35 years and > 35 years, and we compared the patients who did receive a luteal supplementation with triptorelin and those who did not. As shown in Table 3, both pregnancy rates, implantation rates and delivery rates were significantly higher when triptorelin was added, either in women < 35 or > 35 years, with *p* < 0.05.

**Table 3:** Comparison of clinical outcome when data from different regimens of luteal triptorelin were cumulated

No Triptorelin	< 35 years	BP	PR	DR	IR	AR
		87/279 (31,2%)	72/279 (25,8%)	66/279 (23,6%)	17,52%	24,1%
Triptorelin	< 35 years	168/507 (33,1%)	153/507 (30,1%)*	143/507 (28,2%)*	23,45%*	14,8%
No Triptorelin	≥ 35 years	71/241 (29,4%)	61/241 (25,3%)	54/241 (22,4%)	14,12%	23,9%
Triptorelin	≥ 35 years	104/317 (32,8%)	93/317 (29,3%)*	87/317 (27,4%)*	19,78%*	16,3%
No Triptorelin	Total patients	158/520 (30,3%)	133/520 (25,6%)	120/520 (23,1%)	15,6%	24,05%
Triptorelin	Total patients	272/824 (33%)	246/824 (29,8%)**	230/824 (27,9%)**	22,15%**	15,4%

*BP* cycles with HCG positive, *PR* Pregnancy rate (clinical pregnancy), *DR* Delivery rate, *IR* Implantation rate, *AR* Abortion rate (ratio between the pregnancy that did not arrive to delivery and the total HCG positive)

* *P* < 0,05 compared to the corresponding group with no luteal triptorelin

** *p* < 0,01 compared to the corresponding group with no luteal triptorelin

When considering the total number of patients, both under and over 35 years, the high number of patients allowed to reach a significance of *p* < 0.01.

No late OHSS were observed in all the groups of treatment.

## Discussion

In women at risk of developing OHSS, the traditional hCG triggering has been replaced by the use of GnRHa [12], that does not provide the same prolonged stimulation of the corpus luteum. The resulting luteolytic effect, and probably the lack of correct activation of the implantation window, significantly reduces the pregnancy rates [13]. These data on the role of the luteal support determined the development of several methods to improve luteal support, including intensive P and E2 supplementation, mini-hCG doses in repeated administrations, and the “freeze-all” approach [14, 15] .

Starting from the studies on cycles with GnRHa as a trigger, but not only, the need of revisiting the luteal phase in all IVF cycles has gained more evident interest. In 2005, Pirard et al. conducted a feasibility study describing a novel method of luteal-phase support with the use of GnRHa [9]. Because GnRHa induces the secretion of LH, they reasoned that this effect was likely to be preserved throughout the luteal phase in non–down-regulated cycles, thereby providing the necessary luteal-phase support. The administration of midluteal single or multiple boluses of GnRHa in various traditional IVF protocols has gained popularity in recent years. It may be postulated that the beneficial effect of midluteal GnRH supplementation is further augmented by repeated GnRHa administration, as suggested by a recent study of the same group [10]. Fusi et al. also demonstrated that the use of five injections of triptorelin 0,1 mg, one every other day starting from the day of embryo transfer, allowed to rescue the luteal phase in such cycles, avoiding the need of freezing all in most situations, and suggesting us the possibility that triptorelin effect may be beneficial itself for its effects on corpora lutea and endometrium [8].

Different mechanisms seem to be involved in the beneficial effect of GnRHa added to the luteal phase. A meta-analysis of all published data regarding GnRH administration in the luteal phase showed that the implantation rate, the clinical pregnancy rate (CPR) per transfer and the ongoing pregnancy rate were significantly higher in the group of patients who received GnRHa in the luteal phase than in the control group (without the luteal phase GnRHa administration) [7]. The results collected from trials that used GnRH antagonist multi-dose ovarian stimulation protocol highlighted that implantation rate, CPR per transfer and ongoing pregnancy rate were significantly higher in the patients treated with GnRHa in the luteal phase compared with the control group [10]. These findings demonstrate that the luteal phase GnRHa administration may increase both the implantation rate in all stimulated cycles and the CPR per transfer and the ongoing pregnancy rate in cycles that were prepared with GnRH antagonist ovarian stimulation protocol [7, 16–19]. Although the number and morphology of embryos transferred was not different, patients who received GnRH agonist in the luteal phase had higher implantation, ongoing pregnancy and live birth rates than women that did not [4].

It has been hypothesized that GnRH agonist may support the corpus luteum by stimulating the secretion of gonadotrophins from pituitary, or by acting directly on the endometrium through GnRH receptors [9]. It should be noted that GnRH receptors are expressed with greatest intensity during the luteal phase in both the stroma and epithelial cells of the endometrium [20. 21, 22]. Moreover, it has been shown that the administration of a single dose of GnRH agonist in the luteal phase, either Triptorelin of Leuprolide, increases pregnancy, implantation, delivery and birth rates in recipients of donated oocytes in whom ovulation was suppressed, and the corpus luteum was thus absent, suggesting also a direct effect of GnRH agonist on the embryo [3, 6]. The mechanism of action of GnRH agonist on the corpus luteum remains a controversial issue. A number of observational clinical studies reported the consequences of an inadvertent administration of GnRH agonist in the luteal phase. All authors, with only one exception [23], agree that the luteal phase GnRH agonist administration does not compromise the continuation of pregnancy achieved with assisted reproduction procedures, rather it seems to support the implantation [24, 25]. Moreover, a GnRH receptor site was immunolocalized in murine endometrium [26] and a functional LH receptor has been detected in the human uterus [21]. These data suggest that a direct action of GnRH agonist or GnRH agonist-induced LH in the uterine tissues may also be responsible for the effects of GnRH agonist administered in the luteal phase.

The safety of GnRHa at the beginning of pregnancy is still debated in the literature [27, 28]. Preclinical toxicology in non-human animal studies did not indicate any teratogenic effects [27]. Until 1998, more than 340 unexpected spontaneous pregnancies were reported to have been inadvertently exposed to GnRHa administration in the midluteal phase [29]. Among these, a congenital abnormality incidence of 2.5% and a pregnancy loss of 15% was seen, not different from those reported for the IVF and general spontaneous population [29–31]. It should be noted that for many years GnRH depots, such as 3.75 mg Triptorelin, were routinely incorporated in many long protocol ART treatments [32]. In this depot preparation, the active GnRH peptide can be detected in the circulation 6 and 7 weeks after administration [33], exposing the fetus to the peptide for a much longer duration than reported in the present study without any long-term adverse outcomes reported.

Our study was performed on “normal prognosis” patients. We only included cycles when antagonist is used for pituitary suppression for two reasons. First of all, a long agonist cycle has a completely different influence on the endometrium, not comparable to an antagonist protocol, second, the data of luteal administration in such cycles are barely or not supported by literature [34].

The main result that we obtained was that, independently from the scheme used (a single bolus or multiple shots), the addition of triptorelin in the luteal phase increased our main goal, the delivery rate. The power of this statement is given by the number of patients participating to the study, and by being a prospective randomized trial. When all the data are cumulated, the statistic difference between pregnancies and deliveries obtained with or without luteal triptorelin reaches *p* < 0,01, a really important difference, when considering that many factors normally influence the outcome of assisted reproduction techniques. The absence of OHSS in all cycles when triptorelin was added in the luteal phase indicated that its addition does not enhances the OHSS risk.

## Conclusions

In conclusion, we believe that the concept of a simple administration of progesterone in the luteal phase should be revisited. Several ways of improving the luteal phase supplementation can be considered as the low dose HCG or agonists. From our study it appears that triptorelin addition to the luteal phase of antagonist cycles is a good tool to optimize in vitro fertilization results.

## Data Availability

All data generated or analysed during this study are included in this published article.
